# Toll-like receptors are critical for clearance of *Brucella* and play different roles in development of adaptive immunity following aerosol challenge in mice

**DOI:** 10.3389/fcimb.2012.00115

**Published:** 2012-09-07

**Authors:** Jianwu Pei, Xicheng Ding, Yaping Fan, Allison Rice-Ficht, Thomas A. Ficht

**Affiliations:** ^1^Department of Veterinary Pathobiology, Texas A&M University, College StationTX, USA; ^2^Texas AgriLife Research, College StationTX, USA; ^3^Department of Molecular and Cellular Medicine, College of Medicine, Health Science Center, Texas A&M University, College StationTX, USA

**Keywords:** *Brucella*, TLR, MyD88, aerosol infection, innate immunity, adaptive immunity

## Abstract

*Brucella* spp. cause undulant fever in humans and brucellosis in variety of other animals. Both innate and adaptive immunity have been shown to be important in controlling *Brucella* infection. Toll-like receptors (TLRs) represent a group of pattern recognition receptors (PRRs) that play critical roles in the host innate immune response, as well as development of adaptive immunity. In the current report, we investigated the role of TLR signaling in the clearance of *Brucella* and development of adaptive immunity in TLR2^−/−^, TLR4^−/−^, or MyD88^−/−^ mice following aerosol exposure to *B. melitensis* 16 M. Consistent with previous reports, MyD88 is required for efficient clearance of *Brucella* from all three organs (lung, spleen, and liver). The results reveal Th2-skewed immune responses in TLR2^−/−^ mice late in infection and support a TLR2 requirement for efficient clearance of *Brucella* from the lungs, but not from the spleen or liver. Similarly, TLR4 is required for efficient clearance of *Brucella* from the lung, but exhibits a minor contribution to clearance from the spleen and no demonstrable contribution to clearance from the liver. Lymphocyte proliferation assays suggest that the TLRs are not involved in the development of cell-mediated memory response to *Brucella* antigen. Antibody detection reveals that TLR2 and 4 are required to generate early antigen-specific IgG, but not during the late stages of infection. TLR2 and 4 are only transiently required for IgM production and not at all for IgA production. In contrast, MyD88 is essential for antigen specific IgG production late in infection, but is not required for IgM generation over the course of infection. Surprisingly, despite the prominent role for MyD88 in clearance from all tissues, MyD88-knockout mice express significantly higher levels of serum IgA. These results confirm the important role of MyD88 in controlling infection in the spleen while providing evidence of a prominent contribution to protection in other tissues. In addition, although TLR4 and TLR2 contribute little to control of spleen infection, a significant contribution to clearance of lung infection is described.

## Introduction

The genus *Brucella* is a group of Gram-negative, facultative intracellular bacteria that cause brucellosis, a reproductive disease in ruminants, and undulating fever in humans. Brucellosis is one of the most important worldwide zoonotic diseases. Ten species have been identified to date, three of which, including *B. melitensis*, *B. abortus*, and *B. suis* are virulent in humans and represent a significant threat to public health (Atluri et al., 2011). Humans often become infected following inhalation of particles carrying the bacteria or consumption of dairy products contaminated with the organism. Although vaccination is used to successfully reduce the spread of disease, the risk remains high in underdeveloped nations. There are currently no vaccines available that are safe for use in humans, and although generally effective, antibiotic treatments do not always prevent disease recrudescence. As a result of these factors and concern over their potential weaponization, NIH and the CDC/USDA have classified these three species as category B agents.

Both innate and adaptive immunity have been described as contributing to the control of *Brucella* infection (Baldwin and Parent, 2002; Dornand et al., 2002; Baldwin and Goenka, 2006). The role of innate immunity against infection by this pathogen has drawn recent attention as a result of awareness of the role of innate immunity in the establishment of infection and the development of adaptive immunity (Weiss et al., 2005; Oliveira et al., 2011). In contrast, adaptive immunity, including cell-mediated and humoral responses, has been the prominent focus of *Brucella* research over the past few decades.

The innate immune system is composed of a variety of cellular and humoral components, which are the first line of the host defense against invading pathogens. Recognition relies on pattern recognition receptors (PRRs) expressed on/in the cellular components of the innate immune system. Toll-like receptors (TLRs) are the best characterized PRRs. Receptor-ligand interaction via TLRs induces the production of antimicrobial peptides and proinflammatory cytokines through NF-κB, mitogen-activated protein kinase (MAPK) and other signaling pathways (Kawai and Akira, 2006). As a result, TLR signaling is critical to development of the host innate immune response, including recruitment of dendritic cells (DCs) and T effector cells, and upregulation of MHC I and II on antigen presenting cells (APCs) and by extension adaptive immunity against infection. 10 TLRs in human and 13 in the mouse have been identified to date (Kawai and Akira, 2006). TLR2, TLR4, TLR5, and TLR9 recognizing lipopeptide, lipopolysaccharides, flagellin and CpG DNA, respectively, are known to be important in controlling bacterial infections. With the exception of TLR3, the TLRs require the adapter molecule myeloid differentiation factor 88 (MyD88) for signal transduction (Kawai and Akira, 2007). As expected, MyD88 have been shown to be essential for clearance of *Brucella* infection from mice (Weiss et al., 2005; Copin et al., 2007; Macedo et al., 2008).

Several groups have investigated the contribution of TLR signaling to innate immunity against *Brucella* infection in the mouse model. The consensus opinion is that TLR2 is not required to control *Brucella* infection in the mouse (Campos et al., 2004; Copin et al., 2007). However, TLR2 has been shown to be important for cytokine production (Huang et al., 2003; Giambartolomei et al., 2004; Weiss et al., 2005; Macedo et al., 2008; Zwerdling et al., 2008), MHC-II expression (Barrionuevo et al., 2008) and down regulation of the type I receptor for the Fc portion of IgG (FcγRI, CD64) (Barrionuevo et al., 2011) in tissue culture.

The role of TLR4 in *Brucella* infection remains controversial. Some studies suggest that TLR4 is required to control *Brucella* replication in the mouse (Campos et al., 2004; Copin et al., 2007; Macedo et al., 2008); others indicate that TLR4 is not involved (Weiss et al., 2005; Barquero-Calvo et al., 2007). The use of different *Brucella* strains/species in these experiments may account for the observed differences. The influence of the route of infection on the role of TLRs in studies with other pathogens suggests a need to do so with brucellosis, as is the role of TLR-mediated innate immunity in the development of adaptive immune response. These studies employed intraperitoneal (i.p.) inoculation, which is not a typical route for *Brucella* infection. As a result of these reports, the consensus of scientific opinion is that MyD88 contributes significantly to the control of *Brucella* infection while TLR-based signaling plays a lesser role at best. The absence of any role for TLR signaling is consistent with results indicating that the *Brucella* protein TcpB interrupts TLR–based signaling by promoting degradation of MyD88 adaptor like protein, MAL (Chaudhary et al., 2012), and modification of *Brucella* LPS reduces agonist activity (Duenas et al., 2004). However, these studies have been restricted to an atypical route of exposure, and the potential for differential TLR expression associated with different tissues has not been considered (Juarez et al., 2010). Since inhalation represents a major concern to public health, experiments were undertaken to determine the role of TLR signaling in the control of *Brucella* infection following aerosol exposure. The current study, investigates the roles of TLR2, TLR4, and MyD88 in clearance of *Brucella* following respiratory exposure and development of adaptive immune response against *Brucella*. Since the mucosal/respiratory system is the primary portal of entry for human infection documented in many laboratory incidents and relevant to biothreats, it is important to understand the pathogenesis and immune responses resulting from aerosol exposure.

Since TLR signaling is an important component bridging innate and adaptive immunity (Iwasaki and Medzhitov, 2004), failure to clear *Brucella* from organs by TLR signaling deficient mice could be due to defects in the development of adaptive immunity. To test this hypothesis, we determined cellular- and humoral-mediated adaptive immunity in these TLR knockout mice following *Brucella* infection. The results reveal that TLR signaling exhibits significant differences in control and clearance of *Brucella* from selected tissues and in the associated development of an adaptive immune response.

## Materials and methods

### Bacterial strain and growth conditions

The bacterial strain used in these experiments, *B. melitensis* 16 M was obtained from ATCC (#23456) and recovered from an aborted goat fetus. Bacterial cultures were prepared as previously described (Pei and Ficht, 2004). Briefly, *B. melitensis* 16 M was cultured in TSB for 24 h, pelleted by centrifugation at 20,000 ×g, washed with and resuspended in PBS (pH 7.4) at a density of approximately 5 × 10^9^ CFU/ml (Kahl-McDonagh et al., 2007).

### Mouse model of infection

Breeding pairs of TLR2^−/−^, TLR4^−/−^, and MyD88^−/−^ mice were obtained from Dr. S. Akira (Osaka University, Osaka, Japan) via Dr. Michael Berton (University of Texas at San Antonio, San Antonio, TX) and colonies maintained by Comparative Medicine Program (CMP) personnel at TAMU. C57BL/6 control mice were purchased from Jackson Laboratory. All mice were housed in BSL-3 suite in the CMP at Texas A&M University. Mice of both sexes between 8 and 12 weeks old were used in the experiments. Euthanasia was performed using carbon dioxide inhalation. All personnel working with animals received training in rodent handling and euthanasia via the CMP. All animal work was performed in compliance with the Public Health Service (PHS) Policy on Humane Care and Use of Laboratory Animals as described by the National Research Council's Institute for Laboratory Animal Research (ILAR) Guide for the Care and Use of Laboratory Animals.

### Aerosol exposure of mice

Aerosols were generated via nebulization of a *Brucella* suspension (~5 × 10^9^ CFU/ml in PBS) into a Madison Chamber (Madison, Wisconsin) according to the manufacturer's instructions (Kahl-McDonagh et al., 2007). The aerosol chamber was located in a biosafety level 3 (BSL3) facility in the Laboratory Animal Research and Resources (LARR) building staffed and managed by the CMP at Texas A&M University. To assure personal safety, the principal investigator implemented a strict safety protocol, and all procedures were approved by the IBC and IACUC. Following aerosol exposure, one group of mice (*n* = 5) were immediately euthanized via carbon dioxide inhalation and the lungs collected to determine the infecting dose (Kahl-McDonagh et al., 2007). At 1, 2, 4, 6, 8, and 10 weeks after exposure, mice were euthanized and lung, spleen and liver were collected and homogenized in 1 ml each of sterile water containing 0.5% (v/v) Tween-20. One hundred microliters of appropriate dilution of the homogenized tissue were plated on Farrell's selective medium plates followed by incubation at 37°C for at least 3 days. The experimental limit of detection was determined to be 10 CFU per lung, spleen or gram of live, and the results described represent the accumulated data from 4 independent aerosol exposures.

### Lymphocyte proliferation assay

At 8-week post infection (p.i.), one-half of the spleen from each euthanized mouse was used to produce single cell suspensions as previously described. The cell density was adjusted to 2 × 10^6^ per ml using complete RPMI-1640 containing 10% (v/v) heat-inactivated FBS, 1 mM non-essential amino acid, 100 μg/ml of penicillin and 100 U/ml streptomycin. The cell suspension was dispensed to 24-well plates with 1 ml/well and stimulated with heat killed *Brucella* (HKB) or ConA (2 μg/ml) for three days. Following stimulation, supernatants were collected and cytokine synthesis characterized. Cells collected from 100 μl of the suspension were lysed using 1% (v/v) Triton X-100, and the LDH released from live cells was determined using CytoTox 96™ Nonradioactive Cytotoxicity Assay kit (Promega) following the manufacturer's instructions.

### Cytokine ELISA

IFN-γ levels in the culture supernatants were determined 72 h post stimulation using sandwich ELISA kits (PeproTech Inc., Rocky Hill, NJ) according to the manufacturer's instructions (Pei et al., 2008).

### Anti-brucella antibody detection

Blood samples were collected just prior to euthanization from mice at 1, 2, 4, 6, 8, and 10 weeks post exposure. Sera were separated and anti-*Brucella* antibodies, including IgG, IgG_1_, IgG_2a_, IgM, and IgA were evaluated using ELISA. ELISA plates (Nunc) were coated with *B. melitensis* 16 M cell lysate. Briefly, 100 μl of 1 μg/ml lysate in bicarbonate/carbonate buffer (2.93 g NaHCO_3_, 1.59 g Na_2_CO_3_, 0.203 g MgCl_2_ in 1 L of distilled water, pH 9.6) was added to microtiter wells (96 wells/plate) and incubated overnight at 4°C (Pei and Collisson, 2005). Non-specific binding was blocked via incubation with 5% (w/v) non-fat milk in PBS for 1 h at room temperature. The antigen-coated wells were incubated 2 h at room temperature (25°C) with mouse sera diluted in PBS (pH 7.4) containing 0.05% (v/v) Tween-20 (PBS-T) 200 times for IgG1, IgG_2a_, IgM, IgA detection or 1000 times for IgG detection. The plates were then incubated with HRP-conjugated goat anti-mouse immunoglobulins (IgG_1_, IgG_2a_, IgM, IgA, and IgG from Kirkegard-Perry Labs (KPL) for 1 h at room temperature. The wells were washed with PBS-T between steps to remove any unbound materials. Color development followed the addition of 100 μl of TMB substrate KPL. The reaction was terminated by the addition of 50 μl of stop buffer (1 M H_2_SO_4_) and OD_450_ was determined using an ELISA reader (Bio-Rad).

### Statistical analysis

Statistical significance was determined using one-way ANOVA or Student's *t*-test. *P*-values <0.05^*^ and <0.01^**^ were considered to be significant.

## Results

### The role of TLR signaling in clearance of *Brucella* from the lung

**Figure 1:**
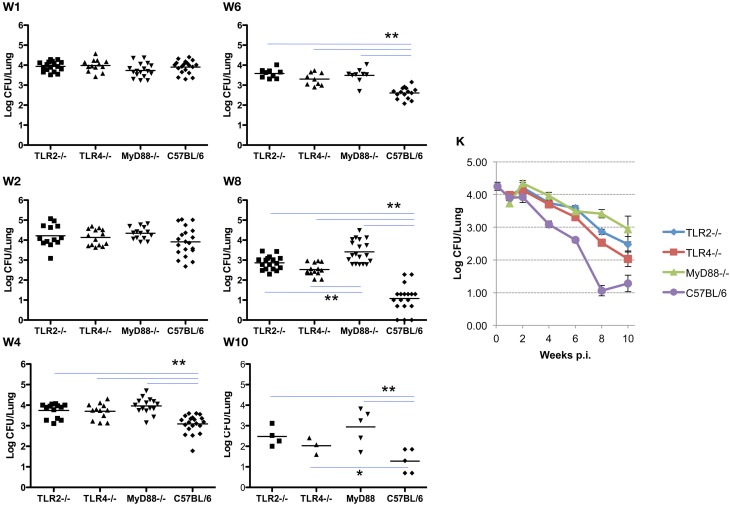
**TLR signaling is required for efficient clearance of *Brucella* from the lungs.** Groups of TLR2^−/−^, TLR4^−/−^, MyD88^−/−^ and C57BL/6 mice were aerosol exposed to *B. melitensis* 16 M. The lungs were collected at weeks 1 (panel **W1**), 2 (panel **W2**), 4 (panel **W4**), 6 (panel **W6**), 8 (panel **W8**), and 10 (panel **W10**) post infection, and bacterial loads were determined. The data were pooled from four separate experiments. The results were analyzed using one-way ANOVA. ^*^P < 0.05, ^**^P < 0.01. The line graph (panel **K**) illustrates the kinetics of bacterial clearance from the lungs. Error bars, S.E.

In these experiments, the infecting dose determined using lungs collected from control C57BL/6 mice (*n* = 5) immediately following challenge is 4.26 ± 0.28 log CFU/mouse, which is consistent with a previous report (Kahl-McDonagh et al., 2007). Bacterial load recovered from the lungs of TLR2^−/−^, TLR4^−/−^, and MyD88^−/−^ mice during the first two weeks following exposure is not significantly different from the C57BL/6 control mice (Figure 1, panels W1–2), indicating that TLR2, TLR4, and MyD88 are not required to clear lung infection early. A significant difference in bacterial burden is detectable by week 4 and extends through week 10, indicating that the contribution of TLR2, TLR4, and MyD88 to the control of *Brucella* infection in the lungs occurs via adaptive immunity (Figure 1). By week 4 p.i., there was a significant difference in the clearance of the organism from each of the knockouts. This pattern persisted through week 10, although significance is lost due to the reduction in the number of animals available at this time point. The absence of MyD88 appears to have a greater impact on the clearance of infection from the lungs over this period, as the bacterial burden in the lungs of MyD88^−/−^ mice is significantly higher than that in TLR2^−/−^ and TLR4^−/−^ mice by week 8 p.i. (Figure 1, panel W8). The kinetics of *Brucella* clearance from the lungs is summarized in Figure 1K, and taken together, these results indicate that TLR2, TLR4, and MyD88 contribute to *Brucella* clearance from the lungs following aerosol challenge.

### The role of TLR signaling in Brucella clearance from the spleen

**Figure 2:**
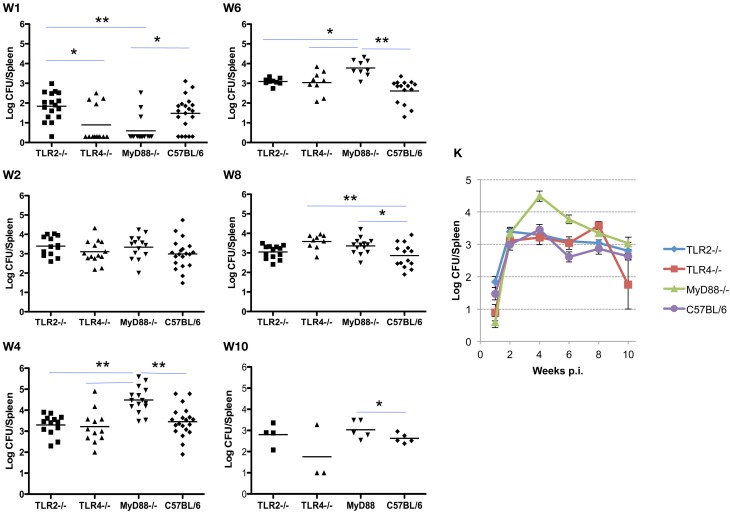
**TLR4 and MyD88 are required to control and clear *Brucella* from the spleens.** Groups of TLR2^−/−^, TLR4^−/−^, MyD88^−/−^ and C57BL/6 mice were aerosol exposed to *B. melitensis* 16 M. The spleens were collected at weeks 1 (panel **W1**), 2 (panel **W2**), 4 (panel **W4**), 6 (panel **W6**), 8 (panel **W8**), and 10 (panel **W10**) post infection, and bacterial loads were determined. The data were pooled from four separate experiments. The results were analyzed using one-way ANOVA. ^*^P < 0.05, ^**^P < 0.01. The line graph (panel **K**) illustrates the kinetics of bacterial clearance from the lungs. Error bars, S.E.

Infection and the kinetics of clearance of *Brucella* from the spleens of mice is notably different from that observed in the lung. Initially, the organism exhibits a delay in systemic distribution to the spleen that requires up to 4 weeks to achieve maximum burden. The delay in systemic spread of infection is evident in the spleen and less dramatically in the liver (see below), with bacterial burden in 9 of 13 spleens from TLR4^−/−^ and 10 of 13 from MyD88^−/−^ mice below the detection limit (10 cfu/spleen). This compares with a delay in only 1 of 17 TLR2^−/−^ mice and 5 of 20 C57BL/6 mice. The MyD88^−/−^ knockout appears to have a reduced ability to control the infection as evidenced by the significant difference in spleen colonization by week 4 (Figure 2, panel W4). Once established infection proceeds similarly, although the MyD88^−/−^ and TLR4^−/−^ mice exhibit a delay in clearance relative to the TLR2^−/−^ and C57BL6 mice (Figure 2, panels W6–10). Systemic spread and the clearance of infection from the spleen is unaffected by the loss of TLR2, with no significant difference observed between the TLR2^−/−/−^ and wild-type mice over the course of infection (Figure 2, panels W1–10). In contrast, both TLR4^−/−^ and MyD88^−/−^ mice exhibited early delays in systemic spread of infection during the first week, which is accompanied by a delay in clearance at later times. Again, the exaggerated decline observed between weeks 8 and 10 in TLR4^−/−^ mice may be an artifact of the reduced animals numbers.

### The role of TLR signaling in Brucella clearance from the liver

**Figure 3:**
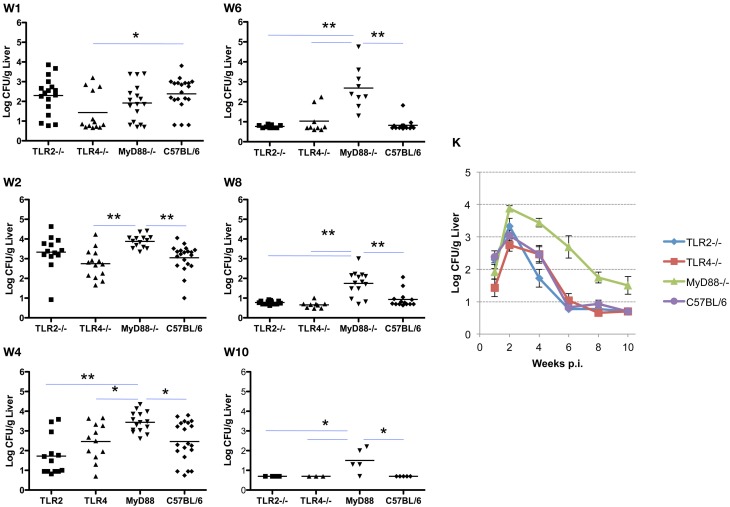
**MyD88 is required for clearance of *Brucella* from the livers.** Groups of TLR2^−/−^, TLR4^−/−^, MyD88^−/−^, and C57BL/6 mice were aerosol exposed to *B. melitensis* 16 M. The livers were collected at weeks 1 (panel **W1**), 2 (panel **W2**), 4 (panel **W4**), 6 (panel **W6**), 8 (panel **W8**) and 10 (panel **W10**) post infection, and bacterial loads were detected. The data were pooled from four separate experiments. The results were analyzed using one-way ANOVA. ^*^P < 0.05, ^**^P < 0.01. The line graph (panel **K**) illustrates the kinetics of bacterial clearance from the lungs. Error bars, S.E.

Similar to the results described for the spleen, systemic spread of the organism to the liver is delayed in TLR4^−/−^ and MyD88^−/−^ mice, and is associated with a corresponding delay in the clearance of infection. Here again, TLR2^−/−^ mice are indistinguishable from control mice in the ability to restrict systemic infection (Figures 2 and 3). As observed in the spleen, the capacity to control infection is most dramatically affected in the MyD88^−/−^ mice (Figure 3, panels W1–10). From week 2 and onward, TLR2^−/−^ and TLR4^−/−^ mice controlled infection and cleared the organisms from the livers indistinguishably from the C57BL/6 mice (Figure 3, panel W10). Taken together, these results reveal that, in the mouse liver; (1) TLR2 and TLR4 are not required to control infection or clearance; (2) the absence of TLR4 reduces accumulation of *Brucella* in the liver, but has little effect on late stage clearance; (3) MyD88 is critical in early control and late clearance of the organism.

### Adaptive cell-mediated immune response following aerosol challenge with Brucella

**Figure 4:**
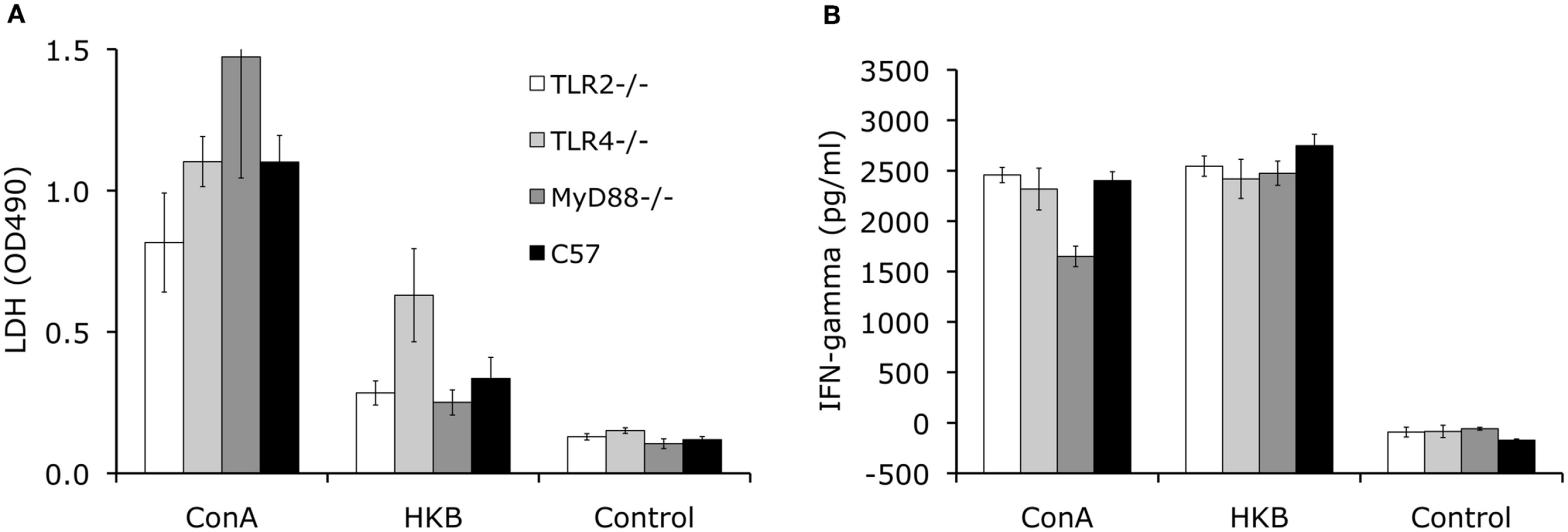
**TLR signaling is not essential for development of cellular-mediated adaptive immunity.** Splenocytes collected from TLR2^−/−^, TLR4^−/−^, MyD88^−/−^ and C57BL/6 mice at 8 weeks p.i. were stimulated with heat killed *Brucella* (HKB) or Con A for 3 days. Lymphocyte proliferation was determined using LDH release assay **(A)**. IFN-γ levels in the culture supernatants were determined via ELISA **(B)**. Data shown was a representative of three independent experiments. Error bars, S.E.

To determine the development of cell-mediated immunity, the splenocytes collected at 8 weeks p.i. from knockout and control mice are stimulated with *Brucella* antigen *ex vivo.* Using an LDH release assay to measure lymphocyte proliferation, no significant differences in proliferation was detected for TLR2^−/−^, TLR4^−/−^, MyD88^−/−^ and control C57BL/6 mice (Figure 4A). This result indicates that cell-mediated adaptive immunity against *Brucella* is unimpaired in these knockout mice. Consistent with the results of the lymphocyte proliferation assay, IFN-γ levels detected in splenocyte supernatants collected from the knockout mice are not significantly different from C57BL/6 mice (Figure 4B).

### Adaptive humoral response following aerosol challenge with Brucella

**Figure 5:**
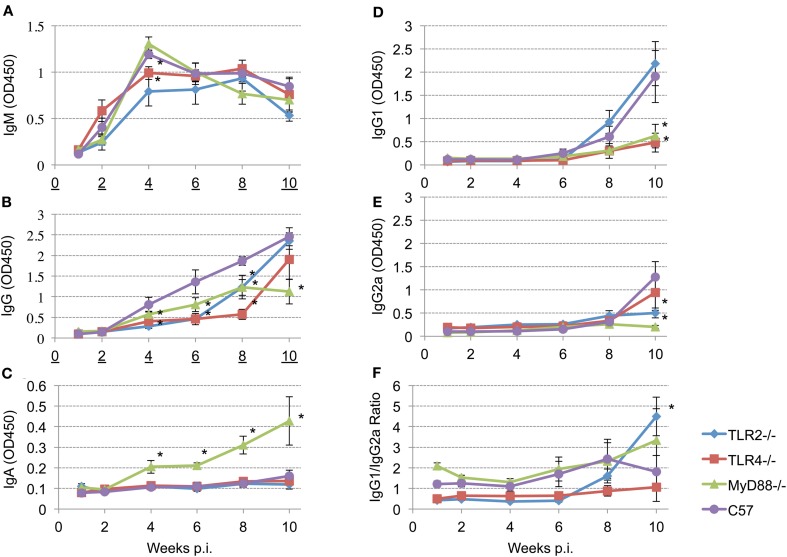
**Different TLRs play different roles in eliciting antibody responses after aerosol challenge.** Groups of TLR2^−/−^, TLR4^−/−^, MyD88^−/−^ and C57BL/6 mice were aerosol exposed to *B. melitensis* 16 M. Blood samples were collected at 1, 2, 4, 6, 8, and 10 weeks post infection and sera were isolated. Immunoglobulin levels IgM (panel **A**), IgG (panel **B**), IgA (panel **C**), IgG1 (panel **D**), IgG2a (panel **E**) in the sera were detected using ELISA. IgG1/IgG2a ratio is illustrated (panel **F**). Error bars, S.E. ^*^P < 0.05 when compared to C57BL/6.

To determine the role of TLR signaling in antibody production and isotype switching, anti-*Brucella* IgM, IgG, IgG_1_, IgG_2a_, and IgA responses were determined following aerosol infection. The results presented reveal detectable levels of IgM by 2 weeks p.i. that peaks by 4 weeks p.i. (Figure 5, panel A). IgM levels begin to decline in the MyD88^−/−^ and C57BL/6 mice at 4 weeks p.i. while, IgM levels in the TLR2^−/−^ and TLR4^−/−^ mice plateau at 4 weeks with a gradual decline by 10 weeks p.i. Any requirement for TLR2 and TLR4 in IgM production is transient (only at 4 weeks p.i.). and IgM levels were never significantly different between MyD88^−/−^ and C57BL/6 mice throughout the experiment.

Total *Brucella*-specific IgG in wild-type mice is detectable starting 4 weeks post-infection (Figure 5, panel B), and increases continuously over the next 6 weeks. In contrast, elevation of the *Brucella* specific IgG levels is delayed in all of the knockout mice. Comparison among the groups revealed that, between weeks 4 and 8 p.i., IgG levels in TLR2^−/−^ and TLR4^−/−^ mice are significantly lower than that observed in the C57BL/6 mice. IgG levels increase in TLR4^−/−^ and TLR2^−/−^ mice by 10 weeks p.i. to levels that are not significantly different from the control C57BL/6 mice. In contrast, IgG levels in MyD88^−/−^ mice exhibit a significant reduction in titer over the course of infection. Serum IgA is not detected in the TLR2^−/−^ and TLR4^−/−^ mice, nor in C57BL/6 mice out to week 10 (Figure 5, panel C). Interestingly, serum IgA is prominent in the sera of MyD88^−/−^ mice and continuously increases over the course of the experiment.

Recent studies have shown that TLRs play a critical role in determining the fate of naive T cells, directing them toward either Th1 or Th2 responses (Dabbagh and Lewis, 2003; Xu et al., 2004). To characterize the role of TLR signaling in determining a Th1 or Th2 response, IgG_1_ (Th2) and IgG_2a_ (Th1) levels and the IgG_1_/IgG_2a_ ratio are calculated following *Brucella* aerosol exposure. IgG_1_ and IgG_2a_ production are above background levels only 8 weeks p.i. (Figure 5, panels D and E). By week 10, TLR2 and C57BL/6 mice produce significantly elevated levels of IgG_1_ (Figure 5, panel D) indicating TLR2 is not required for IgG_1_ production. In contrast, IgG_1_ levels in TLR4 and MyD88 knockout mice are significantly reduced, indicating TLR4 and MyD88 are essential for IgG_1_ production (Figure 5, panel D). Relatively low levels of IgG_2a_ are present at 10 weeks p.i. in all groups, but IgG_2a_ levels in TLR2 and MyD88 knockout mice are significantly reduced relative to control mice. IgG_1_/IgG_2a_ ratios suggesting Th2-skewed immune responses are induced in TLR2^−/−^, MyD88^−/−^ and C57BL/6 mice. The Th2 response in TLR2 knockout mice is significantly elevated compared to wild type mice at 10 weeks p.i. (Figure 5, panel F), indicating TLR2 interactions suppress Th2 response following aerosol infection.

## Discussion

The kinetics of *Brucella spp*. clearance from infected mice differs depending on the genetic background of the strain. In C57BL/6 mice, infection is at or below the limit of detection in the liver as early as 6 weeks p.i. or by 8 weeks in the lung (Figures 1 and 3). In contrast, these same organs in BALB/c mice contain persistent levels of organism 8 weeks p.i. and beyond (Kahl-McDonagh et al., 2007). These results are consistent with previous reports describing enhanced sensitivity of BALB/c mice to *Brucella* infection following i.p. inoculation (Copin et al., 2007), confirming the expected immunological outcome despite the use of different routes of inoculation. One explanation for the improved clearance from C57BL6 mice is generation of a TH1-skewed immune response as opposed to a TH2-skewed response in BALB/c mice (Watanabe et al., 2004).

TLR activation induces the maturation of APCs, enhances antigen presentation, up-regulation of co-stimulatory molecules and cytokine production. Cytokine profiles produced by APCs control CD4^+^ T cell differentiation into either TH1 or TH2 cells. Engagement of different TLRs is expected to affect cytokine production, with significant effect on CD4^+^ T cell differentiation (Dabbagh and Lewis, 2003). Previous studies have shown that *Brucella* infection induces mainly a TH1 response during acute disease (Agranovich et al., 1999; Pasquali et al., 2001; Giambartolomei et al., 2002; Rafiei et al., 2006; Khatun et al., 2009).

It is clear that TLR signaling plays a critical role in the activation of the host innate immune response, including cytokine and chemokine secretion and up-regulation of co-stimulatory molecules in APCs (Kawai and Akira, 2006). Recent studies have shown that these activations indirectly affect the development of adaptive immunity (Iwasaki and Medzhitov, 2010). In addition, it has been demonstrated that functional TLRs are expressed on various T and B cell subsets (Bekeredjian-Ding and Jego, 2009; Booth et al., 2011; Kulkarni et al., 2011). For example, functional expression of TLRs has been extensively investigated on γδ-T cells (Wesch et al., 2011) that have been shown to be important in controlling *Brucella* infection (Bertotto et al., 1993; Ottones et al., 2000). Interaction of TLRs with their respective microbial ligands provides a third signal for B cell activation, which is essential for optimal antigen-specific antibody production and class switch recombination (Bekeredjian-Ding and Jego, 2009; Booth et al., 2011).

*Brucella* are adept at inhibiting the host immune response. *Brucella* virulence derives from expression of an LPS with reduced agonist activity that limits activation of innate immunity and development of an effective adaptive response that promotes invasion and establishment of a replicative niche. In addition, *Brucella* express a protein TcpB (or Btp1) that restricts the proinflammatory response by interfering with MyD88 function. The result is enhanced degradation of MAL (MyD88-adapter-like protein) and restricted expression of NFκ*B.* However, our current results indicate infection varies in different tissues, and that this variation is attributable in part to various PRRs, including TLR2, TLR4, and downstream signaling partners, like MyD88. Interference with TLR signaling by the organism has been described in numerous studies, as has the prominent contribution of MyD88 in limiting infection. The results reported here confirm the importance of MyD88 in the control of infection and extend those findings to include three tissues; spleen, liver and lung. In addition, the experiments performed confirm previous findings concerning the lack of significant contribution of TLR4 and TLR2 with regard to spleen (and liver) infection, but reveal their significant contribution preventing lung infection.

In an effort to explain the control exerted by each of these host factors, an evaluation was made of the corresponding immune response. Since cellular immunity is critical in controlling *Brucella* infection, splenocytes were used to characterize the role of TLR2, TLR4 and MyD88 in the development of cell-mediated adaptive immunity against *Brucella*. Finding that the loss of TLR2, TLR4 and MyD88 had no demonstrable effect on the development of memory T cell response against *Brucella* infection was initially a surprise (Figure 4). However, recent experiments have revealed a disruption of TLR signaling resulting from enhanced degradation of MyD88-adaptor like protein (MAL) that is consistent with previous reports documenting a minimal role for TLR signaling in memory T cell development against other viral and bacterial infections (Way et al., 2003; Fremond et al., 2004; Heer et al., 2007; Seibert et al., 2010; McBride et al., 2011). These results are also in agreement with a recent study showing that IRAK4 (interleukin-1 receptor associated kinase 4), one of the kinases recruited by MyD88 upon stimulation, is not required for generation of CD4+ and CD8+ T cells producing IFN-γ in the late stage of *Brucella* infection (Oliveira et al., 2011). These results suggest that TLR-independent signaling is likely involved in the development of cellular adaptive immunity against *Brucella*.

The contribution of humoral immunity to the outcome of *Brucella* infection is controversial. IgM may enhance opsonic uptake or the activity of complement. However, the differences in IgM production between knockouts are only transitory and have no correlation with differences in immune clearance. In general, IgG levels are significantly reduced in each of the knockout mice. However, the effect in the TLR4 and TLR2 knockouts is transient, while reduced IgG levels are consistently observed in the absence of MyD88 function. A second marker associated with the loss of MyD88 is the significant level of circulating IgA observed in the MyD88 knockout mice. Surprisingly, serum IgA levels were elevated in MyD88^−/−^ mice, but not in TLR2^−/−^ and TLR4^−/−^ mice following *Brucella* infection, suggesting that the IgA class switch recombination or secretion is enhanced in MyD88 knockout mice. Alternatively, higher *Brucella* burden in the lung constantly stimulates IgA secretion. However, this hypothesis is not supported by the fact that serum IgA is not detected in the TLR2 and TLR4 knockout mice despite similar levels of bacterial burden in the lung. In fact, this phenomenon has been previously reported in MyD88^−/−^ mice orally vaccinated with attenuated *Salmonella* expressing a *Streptococcus pneumoniae* surface antigen (Park et al., 2008). It could prove useful to understand the biological mechanism involved and its potential application to vaccine design. The contribution of either elevated IgA or suppressed IgG to the inability of MyD88 knockouts to control infection is under investigation.

Since a reduction in the IgG1/IgG2a ratio is associated with the development of a T_H_1 response and protection against *Brucella* infection, IgG, IgG1 and IgG2a levels were characterized in TLR2, TLR4 and MyD88 following aerosol exposure (Figure 5, panel F). The results reveal a significant contribution of TLR4 and MyD88 to IgG1 production and the importance of TLR2 and MyD88 to IgG2a production. Examination of the IgG1/IgG2a ratio suggests the dependence of a protective T_H_1 response on expression of MyD88 and TLR2, but not TLR4. This is not a totally unexpected result since the *Brucella* LPS, and/or TcpB primarily exert their influence on TLR4-based signaling. The contribution of MyD88 and TLR2 to protective immune response as determined by the reduced bacterial burden in selected tissues is borne out in previous discussion. To reiterate, MyD88^−/−^ exhibits significant delays in clearance from all three tissues examined; TLR2 and TLR4 contribute to clearance from the lung alone.

The importance of TLR signaling in the induction of antibody production remains controversial. In one such study, it was shown that TLR is not required for mice to generate robust antibody response (Gavin et al., 2006). Other researchers report that TLR signaling is critical for antibody production and isotype switching (Pasare and Medzhitov, 2005; Heer et al., 2007). A recent study by Weiss et al. show that TLR2, TLR4, TLR2/TLR4, and MyD88 are not required for anti-*Brucella* IgG production (Weiss et al., 2005). In contrast, our results reveal that production of *Brucella* specific IgG is delayed in TLR2 and TLR4 knockout mice and impaired in MyD88 knockout mice. Possible reasons for the discrepancies include the use of different *Brucella* species, *B. melitensis* 16 M vs *B. abortus* S19, differences in virulence and different routes of inoculation (aerosol exposure vs. intraperitoneal injection). Since IgM production is not affected by MyD88 deficiency and only transiently affected by TLR2 and TLR4 absence, we conclude that TLR2, TLR4 and MyD88 are critical for IgG class switch recombination, which might be critical to the control of *Brucella* infection, and should be considered when *Brucella* designing vaccines.

In our current report, we have clearly demonstrated that TLR2 and TLR4 are critical to clearance of *Brucella* infection from the lung, although less prominently than MyD88. This contrasts significantly with the clearance profile for the spleen and liver and may arise as a result of differences in the TLR expression pattern or levels reported for different tissues (Nishimura and Naito, 2005). It should be noted that based on results of previous studies following i.p. inoculation in which only the spleens were examined, it was concluded that *Brucella* infection was unaffected by the loss of TLR2, however, bacterial load in the lungs and livers were not determined in these studies. (Campos et al., 2004; Weiss et al., 2005; Copin et al., 2007; Macedo et al., 2008). In agreement with previous studies, the results confirm MyD88 as a critical factor in the control of *Brucella* infection in the spleen (Campos et al., 2004; Weiss et al., 2005; Barquero-Calvo et al., 2007; Copin et al., 2007; Macedo et al., 2008). However, caution is warranted in interpretation of the significance of host factors based on the analysis of a single tissue. Recent results in our laboratory indicate that *Brucella* may be recovered from the lung following i.p. inoculation, indicating a need to consider persistence in this tissue irrespective of the route of infection.

Another unexpected observation in this study was the delay in infection of the spleens and livers of TLR4^−/−^ and MyD88^−/−^ mice 1–2 weeks post-inoculation. These results suggest that TLR4 and MyD88 play significant roles in mediating *Brucella* dissemination from the lungs to other tissues, which is consistent with a previous observation indicating a role for TLR4 in the uptake of smooth *Brucella* by macrophage (Pei et al., 2008), and that translocation of bacteria across mucosal or intestinal barriers is mediated by TLR4 (Neal et al., 2006). Evaluation of *Brucella* infection dynamics derived from different routes of entry during the first week post aerosol exposure may be expected to confirm these results and potentially suggest post-exposure treatments.

Over millions of year's co-evolution between host and pathogen have resulted in strategies to survive in the host and cause disease presumably without eliminating the host entirely. Recent studies reveal that bacteria exploit the host TLR pathway, resulting either in enhanced virulence or immune suppression (Arpaia et al., 2011; Round et al., 2011). Our previous study has shown that *Brucella* can use TLR4 to gain entry into the host cells (Pei et al., 2008). It is possible that *Brucella* inhibit IgA class switch recombination or secretion via MyD88 by an unknown mechanism in order to initiate infection through mucosal surfaces.

Overall, our data demonstrate for the first time that TLR2, TLR4, MyD88 are essential for clearance of *Brucella* from the lung following aerosol exposure. Although TLR2, TLR4 and MyD88 are not required for the development of cell-mediated adaptive immunity, they play diverse roles in *Brucella* antigen specific antibody production and antibody class switching. The information obtained from this study will greatly facilitate efforts to understand immunity to *Brucella* and the rational design of novel vaccines against *Brucella* infection.

### Conflict of interest statement

The authors declare that the research was conducted in the absence of any commercial or financial relationships that could be construed as a potential conflict of interest.
